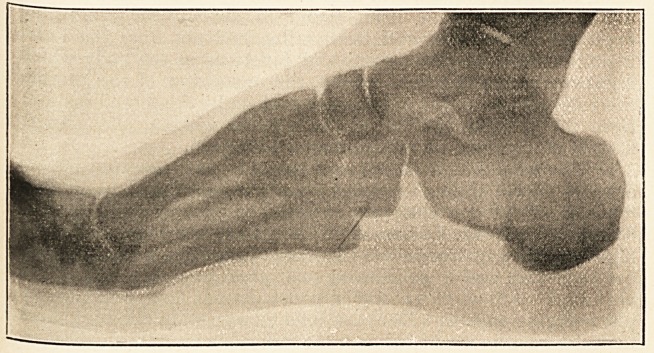# A Case of Needle in Foot Revealed by the X Rays

**Published:** 1896-09

**Authors:** W. E. Pountney


### A CASE OF NEEDLE IN FOOT REVEALED BY THE X RAYS.

BY

W. E. Pountney, M.D.

I report the following case only to show one more instance of the great value and utility of the new photography in aid of diagnosis and treatment in practical surgery.

Mrs. B. consulted me on June 22nd with the following history. When retiring to bed on June 7th she felt a sharp pain in her foot, but did not know the cause until, after search, she found the half of a needle in her stocking. She did not know whether the other portion was in her foot or not. On manipulation I could feel no trace of any foreign body, but on deep pressure in the centre of the plantar surface much pain was produced.

I recommended her to have her foot photographed to establish a diagnosis and to find out if there was any foreign body, and, if so, its immediate locality. She went to University College, Bristol, and there had two skiagrams taken, one from the plantar surface of the foot and one from the side. These showed very plainly the position of the piece of needle. The second of them is here reproduced.

As Mrs. B. did not feel very much inconvenience, she made up her mind to wait a little time before submitting to operation. But she suffered so much pain on June 28th, after a great deal of standing and walking on the previous day, that she decided to have the needle removed. Before operating, I recommended that another skiagram should be taken, to see if the position of the needle had altered. As the new negative showed that it had not moved in the least, ether was administered on July 1st and an incision made along the plantar surface of the foot over the supposed spot where the needle lay, and after some little difficulty and search it was discovered and extracted. The piece measured 9-16ths of an inch in length. The patient made an uninterrupted recovery.

## Figures and Tables

**Figure f1:**